# Molecular Characterization of Ferulate 5-Hydroxylase Gene from Kenaf (*Hibiscus cannabinus* L.)

**DOI:** 10.1155/2013/421578

**Published:** 2013-09-24

**Authors:** Jonggeun Kim, Bosung Choi, Young-Hwan Park, Byoung-Kwan Cho, Hyoun-Sub Lim, Savithiry Natarajan, Sang-Un Park, Hanhong Bae

**Affiliations:** ^1^School of Biotechnology, Yeungnam University, Gyeongsan 712-749, Republic of Korea; ^2^Department of Biosystems and Machinery Engineering, Chungnam National University, Daejeon 305-764, Republic of Korea; ^3^Department of Applied Biology, Chungnam National University, Daejeon 305-764, Republic of Korea; ^4^Soybean Genomics and Improvement Laboratory, US Department of Agriculture, Agricultural Research Service, 10300 Baltimore Avenue, Beltsville, MD 20705, USA; ^5^Department of Crop Science, Chungnam National University, Daejeon 305-754, Republic of Korea

## Abstract

The purpose of this study is to clone and characterize the expression pattern of a *F5H* gene encoding ferulate 5-hydroxylase in the phenylpropanoid pathway from kenaf (*Hibiscus cannabinus *L.). Kenaf is a fast-growing dicotyledonous plant valued for its biomass. F5H, a cytochrome P450-dependent monooxygenase (CYP84), is a key enzyme for syringyl lignin biosynthesis. The full length of the *F5H* ortholog was cloned and characterized. The full-length *F5H *ortholog consists of a 1,557-bp open reading frame (ORF) encoding 518 amino acids (GenBank Accession number JX524278). The deduced amino acid sequence showed that kenaf F5H had the highest similarity (78%) with that of *Populus trichocarpa*. Transcriptional analysis of *F5H* ortholog was conducted using quantitative real-time PCR during the developmental stages of various tissues and in response to various abiotic stresses. The highest transcript level of the *F5H* ortholog was observed in immature flower tissues and in early stage (6 week-old) of stem tissues, with a certain level of expression in all tissues tested. The highest transcript level of *F5H* ortholog was observed at the late time points after treatments with NaCl (48 h), wounding (24 h), cold (24 h), abscisic acid (24 h), and methyl jasmonate (24 h).

## 1. Introduction

 Kenaf (*Hibiscus cannabinus *L.) is an annual dicotyledonous and a fast-growing herbaceous plant, spanning a wide ecological habitat from temperate to tropical areas [1]. Kenaf is regarded as the third largest fiber crop following cotton and jute and is indispensible for pulp and paper industries [2, 3]. Since kenaf has large ranging habitats and fast growth rate, it has great potential for use in biomass production [4–6]. Lignin is an aromatic heteropolymer that is predominantly found in the secondary plant cell walls with cellulose and hemicellulose and is the second most abundant biopolymer on earth after cellulose [7–9]. It provides rigidity to the plant cell wall and hydrophobicity to the vascular system of plants. In addition, lignin is highly associated with the plant defense mechanisms against biotic and abiotic stresses [7–9]. However, lignin is one of the major obstructions in the conversion of plant biomass to pulps, papers, or biofuels [8, 10]. Lignin protects polysaccharides in plant cell walls from microbial degradation. As lignin is strongly bound to polysaccharides, cellulose and hemicellulose polymers are able to get only a limited access to microbial hydrolytic enzymes such as cellulases. For the efficient use of polysaccharides in pulps or biofuels, lignin should be reduced or removed. In addition, the residual products generated during the removal of lignin can inhibit the subsequent processes of saccharification and fermentation. The removal of lignin from plant biomass is a costly process and generates environmental pollutants [10–12]. These shortcomings of the presence of lignin prompted many scientists to develop genetically modified plants with less lignin content or an altered lignin component for its easy degradation [7, 8, 13]. Lignin is synthesized through the phenylpropanoid pathway [14]. There are two major steps of lignin biosynthesis in plants. In the first step, three hydroxycinnamyl alcohol monomers (*p*-coumaryl, coniferyl, and sinapyl alcohols) are synthesized through the phenylpropanoid pathway. In the second step, these alcohol monomers are integrated into the lignin polymers by peroxidase and laccase during polymerization to form *ρ*-hydroxyphenyl (H), guaiacyl (G), and syringyl (S) units, respectively [15, 16]. Among the three units, the S unit is relatively unbranched and has a higher degree of degradability [14]. The biomass of kenaf fibers is favorable for delignification because these fibers contain less amount of lignin and have a high S/G ratio with small amounts of H unit (S 83.3%, G 15.4%, and H 1.3%) [17, 18]. Many attempts have been made to modify the content and composition of lignin in order to increase pulping efficiency. Upregulation of *F5H* increases the S/G ratio [10, 13]. F5H, a cytochrome P450-dependent monooxygenase (CYP84), is specifically required for the synthesis of S lignin [14]. It catalyzes the 5-hydroxylation of coniferaldehyde and/or coniferyl alcohol in the phenylpropanoid pathway. Upregulation of *F5H* in poplar tree was found to increase the S/G ratio that subsequently led to increased pulping efficiency [19]. Conversely, downregulation of *F5H* in alfalfa caused a decrease in the S/G ratio [13]. *Arabidopsis* transgenic lines overexpressing *F5H* showed that it contained S lignin in a larger proportion (92%) [20]. Therefore, F5H is a key enzyme involved in synthesizing monolignol sinapyl alcohol and, ultimately, S lignin moieties. In this study, we cloned and characterized *F5H* ortholog in kenaf to investigate the expression patterns of *F5H* ortholog in various developmental stages, in different tissues, and under various abiotic stresses such as NaCl, wounding, cold, ABA, MeJA, and drought using quantitative real-time PCR (QPCR). 

## 2. Materials and Methods

### 2.1. Plant Materials

Dr. Si-Yong Kang (Advanced Radiation Technology Institute, Korea Atomic Energy Research Institute, Jeongeup, Korea) generously provided kenaf seeds (*Hibiscus cannabinus *L., C-9) that were originally from Russia (GenBank of Korea Rural Development Administration IT number 202789). Seeds were germinated in small pots filled with a sterile nonsoil mixture (Tobietec, Chungbuk, Korea). Kenaf seedlings were grown for up to 4 weeks in a growth room, watered twice a week, and maintained under the following growth condition: 16-h light/8-h dark, 22°C, and 100 *μ*mol m^−2^ s^−1^ light intensity. After 4 weeks in the growth room, kenaf seedlings were transplanted into larger pots (20 cm diameter) filled with nonsoil mixture and were grown in a greenhouse for up to 20 weeks with watering twice a week. Various tissues (roots, stems, leaves, petioles, and flowers) were harvested from 16-week-old kenaf plants during midday of light length. Harvested samples were immediately frozen in liquid nitrogen and stored at −80°C. Leaf tissues were harvested at three development stages: (1) young leaf (YL), <2 cm long; (2) immature leaf (IL), 3–5 cm long; and (3) mature leaf (ML), >9 cm long. In addition, flower samples were harvested at three development stages: (1) young flower (YF), unopened green flower, <2 cm long with green sepal; (2) immature flower (IF), unopened white flower, >3 cm long with green sepal; and (3) mature flower, open white flower.

### 2.2. Stress Treatments

Three-week-old plants, grown in the growth room as previously described, were treated with diverse abiotic stresses. Stem tissues were harvested for RNA extraction. For the wounding treatment, stem tissues were cut twice longitudinally with scissors. For cold treatment, plants were put into a cold room under following growth conditions: 16 h light/8 h dark, 10°C, and 100 *μ*mol m^−2^ s^−1^ light intensity. Plants were treated with ABA (100 *μ*M) or NaCl (200 mM) [21, 22]. Plants treated without ABA and NaCl were used as control samples. Plants were sprayed with 100 *μ*M MeJA dissolved in 0.004% ethanol and then covered with a plastic bag. Plants sprayed with 0.004% ethanol were used as control samples. Stem tissues were harvested 1, 6, 12, 24, and 48 h after treatments. For drought treatment, watering was stopped for 4, 7, 10, and 14 days. For control samples, plants were grown in normal growth conditions with watering once in every 3 days. The harvested samples were frozen in liquid nitrogen and stored at −80°C.

### 2.3. RNA Isolation, Cloning, and QPCR Analysis

Total RNA was isolated from kenaf tissues as previously described [23]. Complementary DNA (cDNA) was synthesized using a Superscript III First-Strand Synthesis Kit (Invitrogen, Carlsbad, CA, USA). Degenerate primers were used to synthesize *F5H* fragment (forward primer, 5′-TT(T/C)TGG(C/A)G(T/G/C/A)CAGATG; reverse primer, 5′-TC(G/A)CT(C/T)GG(C/T)TTCAT-3′). The degenerate primers were designed based on the conserved *F5H* sequences of *Arabidopsis thaliana *(AF068574), *Brassica napus *(DQ679758), *Populus trichocarpa *(AJ010324), *Broussonetia papyrifera *(AY850934), *Eucalyptus globulus *(FJ969838), and *Camptotheca acumina *(AY621153). The amplified product was cloned into the pGEM-T Easy Vector for sequencing (Promega, Madison, WI, USA). Both 5′ and 3′ RACE kits were used to clone a full length of *F5H *ortholog following the manufacturer's instructions (Invitrogen). QPCR was performed as previously described [24] using the Mx3000P QPCR System (Agilent, Santa Clara, CA, USA) with SYBR Green QPCR Master Mix (LPS Solution, Daejeon, Korea). F5H primers used for QPCR were as follows: forward primer, 5′-CTCATTTGCTCCACAGTTTCACT-3′; reverse primer, 5′-TCTATTTCCCTGCAGAGTAGTGTTG-3′. *ACTIN* gene (DQ866836) was used as an expression control: forward primer, 5′-ATGGACAAGTCATTACTATTGGAGC-3′; reverse primer, 5′-AGTGATTTCCTTGCTCATACGGT-3′.

### 2.4. Data Analyses

The following programs were used to design primers and analyze DNA and protein sequences: NCBI Blast (http://blast.ncbi.nlm.nih.gov/), Biology WorkBench (http://workbench.sdsc.edu/), ExPASy Proteomics Server (http://web.expasy.org/compute_pi/) SignalP 4.0 (http://www.cbs.dtu.dk/services/SignalP/), and TargetP V1.1 (http://www.cbs.dtu.dk/services/TargetP/). A phylogenetic tree was constructed from the amino acid sequences by the neighbor joining method using Mega5 (http://www.megasoftware.net/). Duncan's multiple range test was applied to analyze the statistical significance of the differences among means at a significance of *P* ≤ 0.05 using SASS (SASS Inc., Cary, NC, USA). 

## 3. Results

### 3.1. Cloning of a Full-Length *F5H* Ortholog in Kenaf

**Figure 1:**
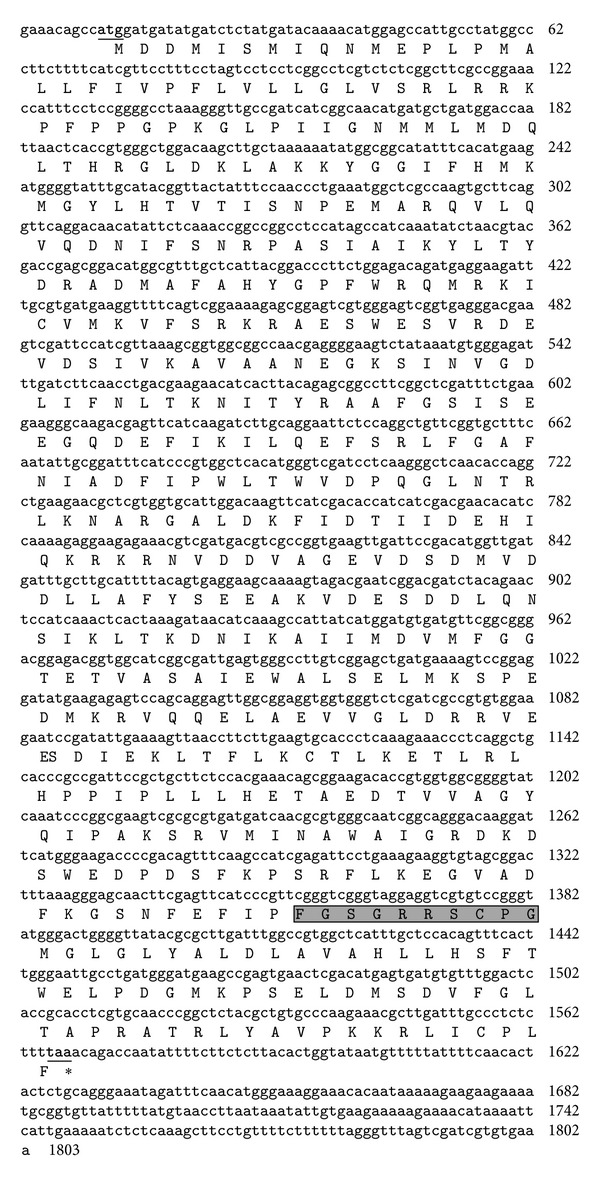
The cDNA and deduced amino acid sequences of *F5H *ortholog from kenaf. The start codon (atg) and stop codon (taa) are underlined and in bold. The gray box indicates the heme-binding ligand (FGSGRRCPG).

**Figure 2:**
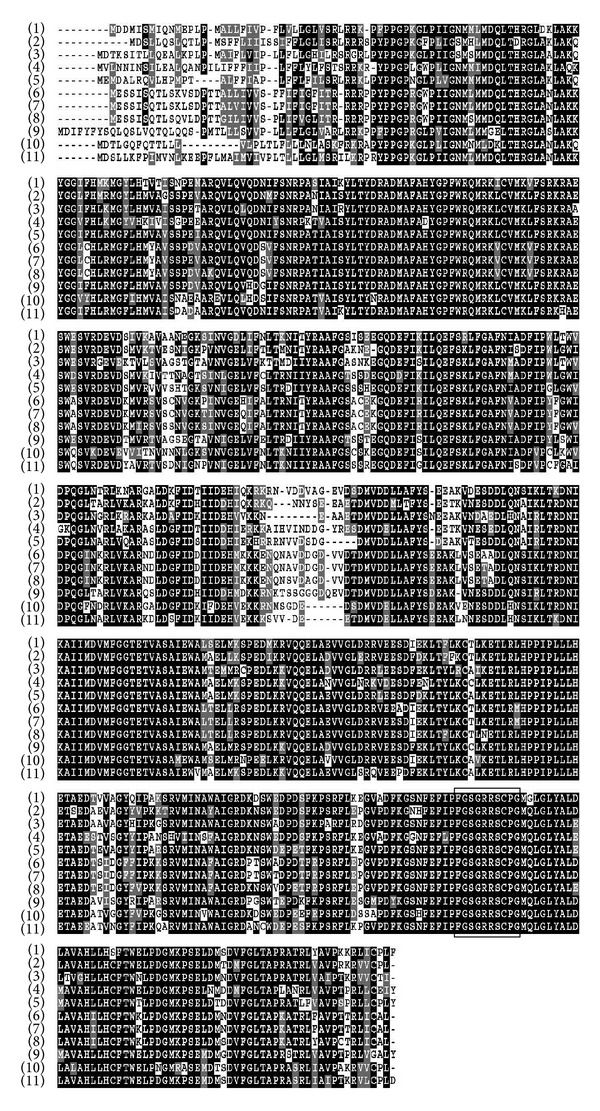
Multiple alignment of F5H sequences. ClustalW and BOXSHADE programs in Biology WorkBench were used to align the sequences. The amino acid residues shaded in black indicate amino acids that are identical with other F5H sequences. The heme-binding ligand (FGSGRRCPG) is boxed. The following F5H sequences were used: (1) *Hibiscus cannabinus *(JX524278), (2) *Populus trichocarpa *(CAB65335), (3) *Broussonetia papyrifera *(AAW50817), (4) *Solanum lycopersicum *×* Solanum peruvianum *(AAD37433), (5) *Camptotheca acuminata *(AAT39511), (6) *Arabidopsis lyrata *(XP002867029), (7) *Arabidopsis thaliana *(CAC26936), (8) *Brassica napus *(ABG73616), (9) *Eucalyptus globulus* (ACU45738), (10) *Medicago truncatula *(XP003629360), and (11) *Medicago sativa *(ABB02161).

**Figure 3:**
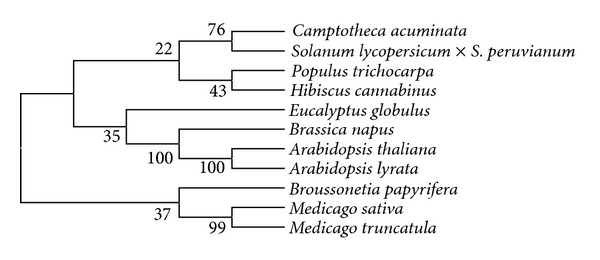
Phylogenic tree of *F5H* orthologs. The phylogenic tree was generated by the neighbor-joining method using ClustalW and Mega5 programs. The numbers at the nodes are bootstrap values from 1,000 replications. The following F5H sequences were used: *Hibiscus cannabinus *(JX524278), *Populus trichocarpa *(CAB65335), *Broussonetia papyrifera *(AAW50817), *Solanum lycopersicum *×* Solanum peruvianum *(AAD37433), *Camptotheca acuminata *(AAT39511), *Arabidopsis lyrata *(XP002867029), *Arabidopsis thaliana *(CAC26936), *Brassica napus *(ABG73616), *Eucalyptus globulus* (ACU45738), *Medicago truncatula *(XP003629360), and *Medicago sativa *(ABB02161).

The full length of kenaf *F5H *ortholog was cloned using degenerate primers and rapid amplification of cDNA ends (RACE) method. Kenaf* F5H *ortholog (GenBank Accession number JX524278) consists of a 1,557-bp open reading frame (ORF) that encodes 518 amino acids (Figure 1). The predicted molecular weight of the deduced protein was 58.62 kDa with an isoelectric point (p*I*) of 5.84 as calculated by the ExPASy Proteomic Server. BlastP analysis revealed that the deduced amino acid sequence of kenaf *F5H* ortholog had high identities with other F5H sequences (Figure 2). The deduced kenaf F5H ortholog shared 78, 78, 77, 77, and 77% identities with F5H sequences from *Camptotheca acuminate *(AAT39511), *Populus trichocarpa *(CAB65335), *Eucalyptus globulus *(ACU45738), *Arabidopsis thaliana* (CAC26936), and *Arabidopsis lyrata *(XP002867029), respectively (Figures 2 and 3). SignalP 4.0 and TargetP V1.1 analyses showed that no signal peptide was detected for subcellular localization in kenaf *F5H* ortholog, which implies cytoplasmic localization. Motif Finder and PROSITE analyses suggested that kenaf *F5H* ortholog may contain a cytochrome P450 cysteine heme-iron ligand signature (FGSGRRSCPG, amino acid position 448–457; Figures 1 and 2), which exactly matched with the previously reported heme-binding ligand in *Arabidopsis* [25]. A phylogenetic tree was constructed from 10 F5H amino acid sequences using Mega 5 program (Figure 3). The phylogenetic tree showed that kenaf *F5H* ortholog had the closest relationship with *Populus trichocarpa* (CAB65335) and was grouped into a subcluster of 4 proteins: *P. trichocarpa*, *Camptotheca acuminate *(AAT39511), and *Solanum lycopersicum* × *Solanum peruvianum* (AAD37433). Cumulatively, all these results emphatically suggest that kenaf *F5H* ortholog is a F5H enzyme. Therefore, kenaf *F5H *ortholog was designated as *HcF5H*.

### 3.2. Expression of *HcF5H* Ortholog in Tissues

**Figure 4:**
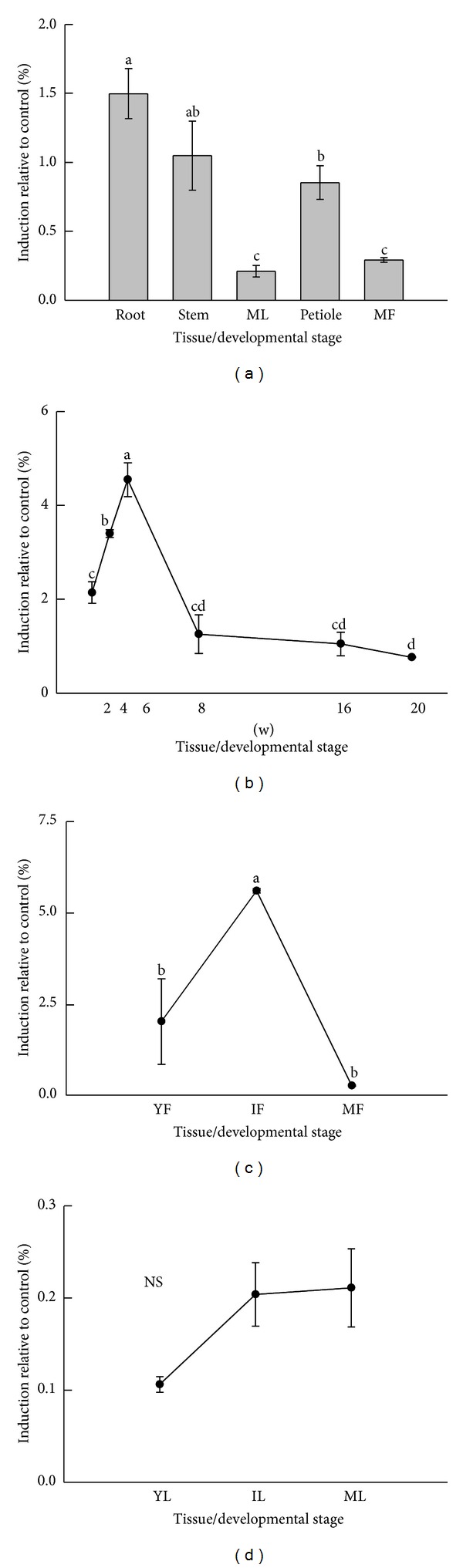
Expression patterns of *HcF5H* ortholog during developmental stages in various tissues. *HcF5H* ortholog was measured using QPCR, and the transcript level was calculated with respect to *ACTIN* level. The percent induction relative to control was calculated after deduction of the control transcript level. (a) *HcF5H* transcript level in various tissues and organs from 16-week-old plants, (b) *HcF5H* transcript level during stem development (2, 4, 6, 8, 16, and 20 weeks after sowing), (c) *HcF5H* transcript level during flower development (YF: young flower; IF: immature flower; MF: mature flower), and (d) *HcF5H* transcript level during leaf development (YL: young leaf; IL: immature leaf; ML: mature leaf). The vertical bars indicate means ± standard error of three biological replications. The letters on top of each point indicate significant differences between the mean values (*P* ≤ 0.05). NS: not significant; w: week.

The expression patterns of* HcF5H* transcript were analyzed in various tissues and organs including roots, stems, leaves, petioles, and flowers using QPCR. *HcF5H *transcript was found to be ubiquitous in all the tested samples (Figure 4(a)). A high level of *HcF5H* transcript expression was observed in root, stem, and petiole tissues of 16-week-old kenaf plants. *HcF5H* was highly expressed in young stems (6 week old, 4.54% relative *ACTIN*; Figure 4(b)) and immature flowers (5.61% relative *ACTIN*; Figure 4(c)), while the transcript level was not significantly induced during leaf development (Figure 4(d)). During stem development, *HcF5H* transcript showed a significant increase at 6 weeks and then sharply decreased for up to 20 weeks. During flower development, the highest transcript level of *HcF5H* was observed in immature flowers, while low level of the transcript was found in young and mature flowers. The transcript values during developmental stages in all tissues were significant (*P* ≤ 0.05), except for the transcripts in leaf development (*P* > 0.05). 

### 3.3. Expression of *HcF5H* Ortholog in Response to Various Abiotic Stresses

**Figure 5:**
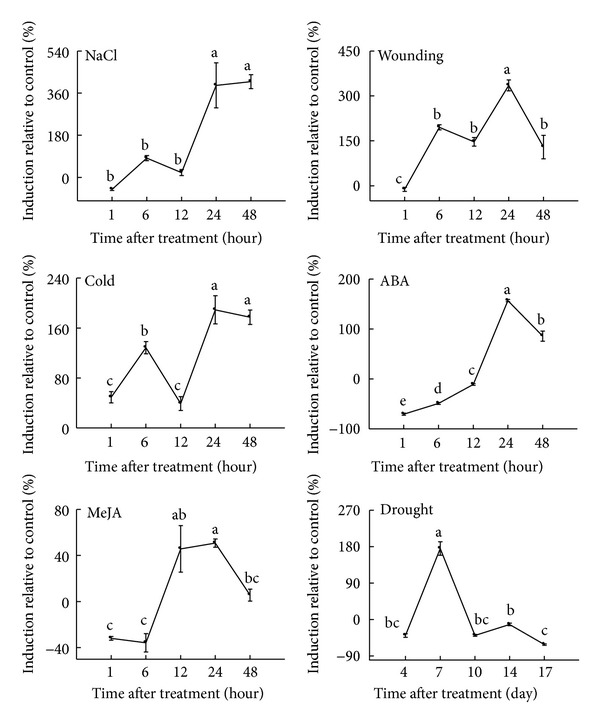
Expression patterns of *HcF5H* ortholog in response to various abiotic stresses. Abiotic stresses were applied to 3-week-old plants, and stem tissues were harvested for QPCR analysis. *HcF5H* ortholog was measured using QPCR, and the transcript level was calculated with respect to *ACTIN* level. The percent induction relative to control was calculated after deduction of the control transcript level. The vertical bars indicate means ± standard error of three biological replications. The letters on top of each point indicate significant differences between the mean values (*P* ≤ 0.05).

*HcF5H* expression patterns were analyzed using 3-week-old stem tissues in their response to various abiotic stresses (NaCl, wounding, cold, ABA, MeJA, and drought) using QPCR. *HcF5H *transcript was induced by all treatments with the highest responses to NaCl and wounding treatments (Figure 5). The highest induction of *HcF5H* was observed at 24–48 h after NaCl treatment. In wounding treatment, a biphasic expression was observed. *HcF5H* was induced at 6 h after the treatment, declining marginally by 12 h. The secondary induction was observed at 24 h after treatment, which was the highest in wounding treatment. The transcript was sharply downregulated later on. Biphasic expression was also observed in cold treatment, in which *HcF5H* transcript was upregulated at 6 h after treatment and then sharply downregulated at 12 h. The secondary induction was observed at 24 h, when the highest transcript level was occurred. When kenaf was treated with ABA and MeJA, *HcF5H *transcript reached its maximum level at 24 h after treatment. In ABA treatment, the transcript level increased up to 24 h after treatment and showed a decrease at 48 h. In the case of MeJA, the transcript level was sharply increased at 12–24 h after treatment and then decreased at 48 h. *HcF5H* transcript level was maximum 7 days after watering was stopped and then dramatically decreased. All transcript values in response to various abiotic stresses were statistically significant based on Duncan's multiple range test (*P* ≤ 0.05).

## 4. Discussion

### 4.1. Characteristics of *HcF5H*

F5H enzyme is a cytochrome P450-dependent monooxygenase (P450s) that catalyzes the 5-hydroxylation of coniferaldehyde and/or coniferyl alcohol in the phenylpropanoid pathway [15]. P450s are a large group of heme-containing enzymes that generally catalyze NADPH- and O_2_-dependent hydroxylation reactions. In plants, two P450s catalyze reactions of the general phenylpropanoid pathway: cinnamate 4-hydroxylase (C4H) and ferulate 5-hydroxylase (F5H). While C4H belongs to P450 CYP73 family, F5H is a member of the CYP84 family of P450s [25]. *Arabidopsis* F5H was first defined as CYP84 and two F5H homologs (AtF5H1 and AtF5H2) were identified in the *Arabidopsis* genome [25]. AtF5H1 is now well characterized in many plants including *Arabidopsis*, *Liquidambar styraciflua*, and *Brassica napus* [25–27]. AtF5H2 is a more divergent member of the CYP84 family, and no genes that closely resemble AtF5H2 are found in other plants [28]. Three F5H homologs were identified in *Brassica napus *(BnF5H1, BnF5H2, and BnF5H3) [27]. BnF5H1 and BnF5H2 are very similar to each other (98% identity at nucleotide sequence and 99% identity at amino acid sequence) and show high identity with AtF5Hs (90% identity at amino acid sequence and 93% identity at amino acid sequence). Therefore, BnF5H1 and BnF5H2 are thought to belong to one group. BnF5H3 also shows the similar level of identity to the corresponding portion but lacks a codon for Proline at position 39, which is present in other F5H genes [27]. These two groups of BnF5H therefore must have been formed before the divergence. Also, the divergence between either BnF5H1 or BnF5H2 and BnF5H3 suggests that the duplication may have occurred very early in the lineage of the *Brassica *spp., whose ancestor has not been identified yet [29]. Only one putative *HcF5H* gene was isolated in this study, but we suspect there might be more than one in kenaf. The deduced amino acid sequence of *HcF5H* showed 74–78% identity with F5H sequences of other plants (Figure 2). Phylogenic tree showed that *HcF5H* shared amino acid sequence identity with other plant F5Hs (Figure 3). *HcF5H* may not contain a significant signal peptide, and so it is thought to be a cytoplasmic protein. In previous studies, however, AtF5H1 and AtF5H2 were found to contain fully conserved membrane anchor regions, and AtF5H2 was expected to have an ER-targeting peptide [28]. In our study, we identified that *HcF5H* contains a cytochrome P450 cysteine heme-iron ligand signature (FGSGRRSCPG), which is homologous with the previously reported heme-binding domain that is well conserved in the CYPs of higher plants [30]. In addition, alignment of seven proteins of the CYP73 gene family showed similar results with the previous study [31]. Therefore, we conclude that *HcF5H* may function as an F5H enzyme.

### 4.2. Significant Difference in *HcF5H* Expression in Different Tissues and Developmental Stages

QPCR analysis revealed that *HcF5H* was expressed in all tissues tested (Figure 4(a)). *HcF5H* was not regulated during leaf development (Figure 4(b)). The highest expression of *HcF5H* was detected in immature flowers and young stems (Figures 4(c) and 4(d)). These results resemble the expression profile of *AtF5H *in *Arabidopsis* [28]. In *Arabidopsis*, both *AtF5H1* and *AtF5H2* were present in all tissues, with stem tissues expressing them at a high level. Moreover, *AtF5H2* had the highest expression in the early stage of stem development. Most abundant transcript level of *BnF5H* was also detected in the stem tissues of *B. napus* [27]. In kenaf, the highest expression level of *HcF5H* was detected in young stem during stem development. The F5H-deficient mutant (*fah1*) accumulated only guaiacyl lignin, while *F5H-*overexpressing transgenic plant produced lignin with mostly syringyl propane units [20]. These results imply that *F5H* expression is essential for S lignin synthesis during stem development. In contrast to the previous studies, *HcF5H* showed the highest expression level in immature flower tissues in our study. In flowers and seeds, *F5H* is required for the production of sinapate esters, which are intermediates of syringyl lignin biosynthesis in higher plants, and some of them serve as precursors for important secondary metabolites [32]. For example, phenylpropanoid-derived compounds such as sinapate and flavonoids were detected at high levels in *Arabidopsis* flowers [33]. In addition, disruption of *AtF5H *led to a limited production of lignin, which resulted in a dwarf floral architecture in the *f5 h *mutant [34]. Therefore, F5H is an important enzyme in flower development. In this study, *HcF5H *expression was found to be significantly induced during flower development, reaching its maximum level in the immature flower. Overexpression of *AtF5H* in *AtCOMT*-deficient *Arabidopsis* caused the accumulation of 5H monomers [35]. These 5H monomers were either directly or indirectly detrimental to pollen wall-forming sporopollenin biosynthesis, which is thought to protect pollens from UV radiation and desiccation. The overexpression of *HcF5H* during flower development could be explained by the pollen wall formation. Overall, expression patterns of *HcF5H* strongly point toward the possibility that this putative enzyme may function as F5H in kenaf.

### 4.3. Significant Induction of *HcF5H* in Response to Various Abiotic Stresses

Plants are subjected to various stress conditions during their life time. They have a generic signal transduction pathway that starts with signal perception, followed by the generation of second messengers such as calcium, reactive oxygen species (ROS), and inositol phosphates [36]. These biotic and abiotic stresses can turn on a plant's defense mechanisms, which include the production of various defensive enzymes and proteins as well as cell wall reinforcement through lignin deposition [23, 37–39]. Lignin induction has been correlated with cold, drought, or light stresses as well as mechanical injuries in plants [23]. Although lignin biosynthesis itself is well characterized, the mechanism regulating ectopic deposition of lignin in response to environmental stimuli is not well understood. Environmental stresses can activate the synthesis of phytohormones in plants [40, 41]. These phytohormones can produce the initial signals for the gene regulation in lignin biosynthesis and others. The initial signals subsequently produce a second-round signaling pathway. In previous studies, we showed that kenaf genes involved in lignin biosynthesis were controlled by various environmental stresses and phytohormones [42–46]. However, this is the first report that showed the upregulation of *HcF5H* as a result of various abiotic stresses in kenaf. *HcF5H* is found to be induced in response to treatments that include wounding, cold, drought, and additions of NaCl, ABA, and MeJA (Figure 5).

*NaCl*. Soil salinity poses a severe environmental restriction limiting the availability of land for sustainable agriculture [47]. Many scientists have identified the genes that cause salt tolerance, so that the effects of salinity in agriculture can be overcome. According to previous reports, increased lignification or altered monolignol composition in plant cell walls provides an effective way to overcome salinity stress [48]. In this study, *HcF5H* was highly induced by NaCl (Figure 5). Similar result was reported in *Brassica juncea *[49]. Moreover, NaCl treatment also induced the genes encoding *S*-adenosyl-l-methionine synthase (SAMS) in tomato, *O*-methyltransferase (COMT) in *Tamarix hispida*, and phenylalanine ammonia-lyase (PAL), *ρ*-coumarate 3-hydroxylase (C3H), and caffeoyl-coenzyme A 3-*O*-methyltransferase (CCoAMT) in kenaf [42, 44, 45, 50, 51]. 

*Wounding.* Wounding has proved to be one of the most effective ways by which control of phenylpropanoid biosynthesis can be understood [23, 52, 53]. For example, wounding induces the genes involved in lignin biosynthesis, such as *PAL *(phenylalanine ammonia-lyase**)**, *C4H (cinnamate 4-hydroxylase)*, *F5H*,* CAD *(cinnamyl alcohol dehydrogenase),* CCR* (cinnamoyl-CoA reductase), and *4CL* (4-coumarate-CoA ligase), which caused accumulation of lignin compounds surrounding the wounding sites [23, 52, 53]. Wounding also induced the synthesis of lignin precursors and ferulate esters in *Solanum tuberosum* [54]. QPCR analysis suggests that *HcF5H* is highly induced by wounding (Figure 5). These results point to the fact that plants enhance lignin production in order to protect them from wounding through the regulation of the genes involved in lignin biosynthesis such as *F5H*.

*Cold.* Low temperature causes an alteration in plant lignin contents [23]. The adaptation of plants to low temperature is thought to involve a genetic response controlling the physical and biochemical changes. It was reported that poplar seedlings grown at 10°C showed an increase in lignin content [55]. Cold treatment induced the accumulation of phenylpropanoid lignin precursors such as *ρ*-coumaric acid, ferulic acid, and synaptic acid in *Brassica napus *[56]. A total 3,379 genes showed altered expression patterns during cold acclimation in *Arabidopsis* [57]. As a key enzyme in lignin synthesis, *F5H* induction by cold might lead to lignification in kenaf stem tissues. It has been reported that cold treatment can upregulate the genes involved in lignin synthesis such as *PAL*, *CCoAOMT*,* C3H*,* HCT*, and* COMT1* in kenaf stem tissues [42–46].

*ABA.* The universal stress hormone ABA is substantially induced during a plant's response to abiotic stress [58]. When the salt stress signal is received by plants, ABA synthesis is induced, which triggers other signaling processes such as the accumulation of calcium ions [59]. ABA is an important phytohormone and plays a critical role in response to stresses including drought, cold, and osmotic stress [60, 61]. Studies suggest that two types of ABA signal transduction pathways are present in plants: one is ABA-independent, and the other is ABA-dependent [60]. The genes involved in ABA biosynthesis were induced by drought and cold treatments, and they were not responsive to exogenous ABA treatment [60]. When detached leaves were treated with ABA, *F5H* was found to be induced in *Camptotheca acuminata* [62], suggesting that *F5H* might belong to the ABA-dependent pathway. Similar response pattern was observed when *HcF5H* was treated with ABA, indicating that *HcF5H* can be categorized into an ABA-dependent pathway. Similar induction patterns were observed for treatments with ABA and NaCl.

*MeJA.* Exogenous application of MeJA caused the accumulation of lignin, phenolic compounds, and defense enzymes in eggplant roots [63]. Jasmonic acid (JA) or its methyl ester jasmonate acts as an important regulator of responses to wounding and environmental stresses [64]. *CaF5H *transcript was induced by MeJA treatment in* Camptotheca acuminate* [62]. In our study, we found the upregulation of *F5H* by MeJA in kenaf. This result strongly suggests that MeJA-dependent pathway regulates wound-induced *F5H* activation similar to other *F5H* orthologs. However, MeJA treatment only weakly induced *HcF5H* compared to other treatments. Similar result was observed in the expression of *CaF5H* [62]. It is thought that exogenous MeJA alone might not be effective in mediating the expression of *F5H*. Other factors such as ABA and H_2_O_2_ are also required for stronger expression. 

*Drought.* In this study, drought induced *HcF5H* expression at early time point after watering was stopped (7 days). *HcF5H* sharply dropped to initial levels later. The level of lignin deposition and gene expression may be dependent on plant tissues and drought period. In a previous study, there was a reduction in the amount of ferulic acid and an induction of *ρ*-coumaric and caffeic acids in maize xylem sap 12 days after watering was stopped [65]. Previous studies reported the induction of genes involved in lignin biosynthesis [23, 42–45]. During the drought period, plant cell wall becomes impermeable to water due to the accumulation of lignin, which causes the reduction in water transpiration [66]. Therefore, it is thought that lignification is one of the primary responses to drought stress. 

In summary, a full length of the gene *F5H* encoding ferulate 5-hydroxylase was cloned from kenaf (*Hibiscus cannabinus *L.). Transcriptional and phylogenetic analyses indicated that *HcF5H* ortholog belonged to known *F5H* genes of plants. On the transcript level, *HcF5H* was regulated by the developmental stages in various tissues and in response to various abiotic stresses. These results suggest that *HcF5H* may play important roles in kenaf plant for better adaptation to environmental stresses.
